# Three Epidemics of Invasive Multidrug-Resistant *Salmonella* Bloodstream Infection in Blantyre, Malawi, 1998–2014

**DOI:** 10.1093/cid/civ691

**Published:** 2015-10-07

**Authors:** Nicholas A. Feasey, Clemens Masesa, Chikondi Jassi, E. Brian Faragher, Jane Mallewa, Macpherson Mallewa, Calman A. MacLennan, Chisomo Msefula, Robert S. Heyderman, Melita A. Gordon

**Affiliations:** 1 Liverpool School of Tropical Medicine, United Kingdom; 2 Malawi Liverpool Wellcome Trust Clinical Research Programme; 3 University of Malawi College of Medicine, Blantyre; 4 The Jenner Institute, Nuffield Department of Medicine, University of Oxford; 5 Wellcome Trust Sanger Institute, Wellcome Trust Genome Campus, Hinxton, Cambridge; 6 Division of Infection and Immunity, University College London; 7 Institute for Infection and Global Health, University of Liverpool, United Kingdom

**Keywords:** *Salmonella*, bloodstream infection, Africa, multi-drug resistance

## Abstract

***Background.*** The Malawi Liverpool Wellcome Trust Clinical Research Programme (MLW) has routinely collected specimens for blood culture from febrile patients, and cerebrospinal fluid from patients with suspected meningitis, presenting to Queen Elizabeth Central Hospital (QECH), Blantyre, Malawi, since 1998.

***Methods.*** We present bloodstream infection (BSI) and meningitis surveillance data from 1998 to 2014. Automated blood culture, manual speciation, serotyping, and antimicrobial susceptibility testing were performed at MLW. Population data for minimum-incidence estimates in urban Blantyre were drawn from published estimates.

***Results.*** Between 1998 and 2014, 167 028 blood cultures were taken from adult and pediatric medical patients presenting to QECH; *Salmonella* Typhi was isolated on 2054 occasions (1.2%) and nontyphoidal *Salmonella* (NTS) serovars were isolated 10 139 times (6.1%), of which 8017 (79.1%) were *Salmonella* Typhimurium and 1608 (15.8%) were *Salmonella* Enteritidis. There were 392 cases of NTS meningitis and 9 cases of *Salmonella* Typhi meningitis. There have been 3 epidemics of *Salmonella* BSI in Blantyre; *Salmonella* Enteritidis from 1999 to 2002, *Salmonella* Typhimurium from 2002 to 2008, and *Salmonella* Typhi, which began in 2011 and was ongoing in 2014. Multidrug resistance has emerged in all 3 serovars and is seen in the overwhelming majority of isolates, while resistance to third-generation cephalosporins and fluoroquinolones is currently uncommon but has been identified.

***Conclusions.*** Invasive *Salmonella* disease in Malawi is dynamic and not clearly attributable to a single risk factor, although all 3 epidemics were associated with multidrug resistance. To inform nonvaccine and vaccine interventions, reservoirs of disease and modes of transmission require further investigation.

Malawi is a small (118 000 m^2^), subtropical country located in sub-Saharan Africa. There is one long rainy season from November to March [1].

It is consistently rated as one of the 10 poorest countries in the world. The estimated mean per capita income in 2013 was $270 and 90% of people live on <$2/day (http://www.worldbank.org/en/country/malawi). The population at the time of the last census in 2008 was approximately 13 million; however, with a fertility rate of 5.3 births per woman, sharp falls in under-5 mortality, and increasing adult life expectancy, this is projected to rise to 16.3 million by 2015 (www.nsomalawi.mw). Blantyre is the principal city in the southern region, with an urban population of approximately 885 000 (www.nsomalawi.mw), and Blantyre district has a population of 1.3 million.

Disease due to serovars of *Salmonella* is commonly considered to be either typhoidal (also known as enteric fever) due to human-restricted invasive *Salmonella enterica* serovars Typhi and Paratyphi A, or nontyphoidal (NTS), due to one of the other *Salmonella* serovars, and commonly associated with self-limiting enterocolitis. Invasive nontyphoidal *Salmonella* (iNTS) disease was first reported in Blantyre as a cause of life-threatening disease in children in 2000 in association with human immunodeficiency virus (HIV), severe malaria, severe anemia, and malnutrition [2–4]. Subsequently, iNTS disease has been reported in adults in Blantyre in association with advanced HIV infection [2]. The national prevalence of HIV in adults was estimated at 10.3% in 2013 (www.unaids.org), but at 22% in urban Blantyre in 2001, falling to 17.9% in 2010 [5]. There has been a highly successful antiretroviral therapy (ART) program, which has been extensively rolled out since 2005. In 2013, the total number of patients alive on ART in Malawi was 472 865, representing coverage of 83% of patients with a CD4 count ≤350 cells/µL (www.unaids.org).

Malawi is a malaria-endemic country with seasonal peaks; the malaria season commences shortly after the start of the rains and lasts until May/June. All febrile children presenting to Queen Elizabeth Central Hospital (QECH), Blantyre, have a thick film examined for malaria parasites; between 2001 and 2010, 61 320 of 242 953 (25%) patients were positive for *Plasmodium falciparum*. Numbers of positive slides declined after 2003, and then leveled off up to 2010 [6].

The prevalence of underweight-for-height status in children aged <5 years in Malawi, which was static between 2000 and 2004, has declined from 6% in 2004 [7] to 4% in 2010 [1]. Admissions for severe acute malnutrition also peak during the rainy season, as increased infection risk coincides with the peak of the “hungry season” when household food supplies are running low while the new season's crops are growing.

Older antimicrobials, such as amoxicillin, chloramphenicol, and cotrimoxazole have been widely available in the community for many years. Cotrimoxazole prophylactic therapy is widely used for patients with HIV infection, and sulfonamides have also been extensively used to treat malaria as part of the combination agent sulfadoxine-pyrimethamine since 1993. Ciprofloxacin (2002) and ceftriaxone (2005) were more recently introduced and are more difficult to acquire outside large government hospitals.

## METHODS

### Study Site

QECH is the largest government hospital in Malawi and serves Blantyre district and the Southern region, providing free healthcare to approximately 10 000 adult (age ≥16 years) and 50 000 pediatric medical inpatients a year, of whom an estimated 70% of adult inpatients are HIV infected [8]. Diagnostic microbiology facilities were provided by the Malawi Liverpool Wellcome Trust Clinical Research Programme (MLW; http://www.mlw.medcol.mw/).

### Time Period

**Figure 1.**
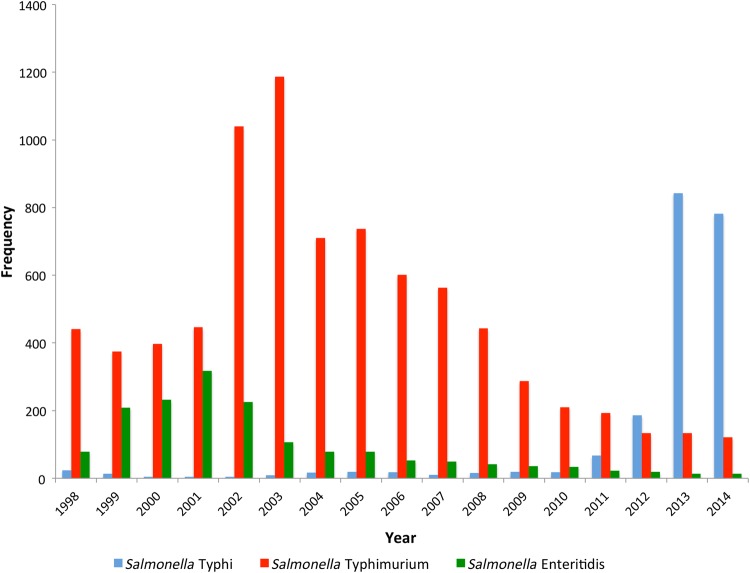
Temporal trends in *Salmonella* Enteritidis, *Salmonella* Typhimurium, and *Salmonella* Typhi bloodstream infection at Queen Elizabeth Central Hospital, Blantyre, Malawi, 1998–2014.

MLW instituted routine blood culture in 1998 and routine cerebrospinal fluid (CSF) culture in 2000. We extracted data from passive hospital-based longitudinal surveillance for bloodstream infection (BSI) from 1998 through 2014 and for meningitis from 2000 through 2014 (Figure 1).

### Eligibility Criteria

During the study period, blood cultures were routinely obtained from any adult medical patient with an axillary temperature >37.5°C or clinical suspicion of sepsis. Blood cultures were obtained from febrile children admitted to the hospital who had a thick blood film that was negative for malaria parasites or who were considered to be critically ill regardless of the thick-film result or temperature. CSF was routinely obtained from all patients in whom meningitis was clinically suspected.

### Estimation of Denominator Population and Minimum Incidence of *Salmonella* BSI

Population projections following the 2008 census were used to provide a denominator for minimum incidence calculations (National Statistics Office, Malawi; www.nsomalawi.mw/2008-population-and-hou​sing-census.html). Minimum incidence of invasive *Salmonella* per 100 000 patients per year was calculated using an estimated sensitivity of blood culture of 50%; therefore, the number of positive blood cultures recorded in each year was doubled to provide a numerator for each of typhoid fever and iNTS disease [9, 10].

### Age Distribution

**Figure 2.**
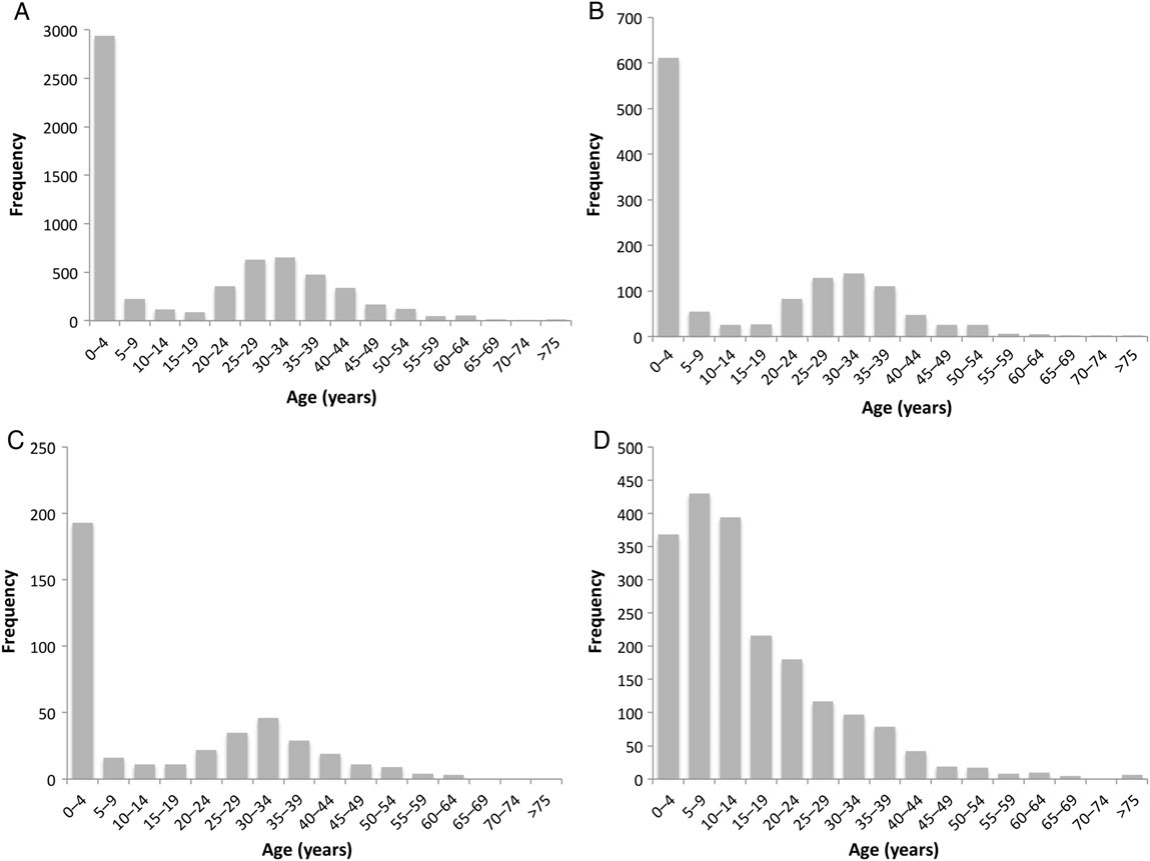
Frequency plots of aggregate age distribution data for *Salmonella* Typhimurium (*A*), *Salmonella* Enteritidis (*B*), *Salmonella* species (*C*), and *Salmonella* Typhi (*D*) in 5-year age groups.

**Figure 3.**
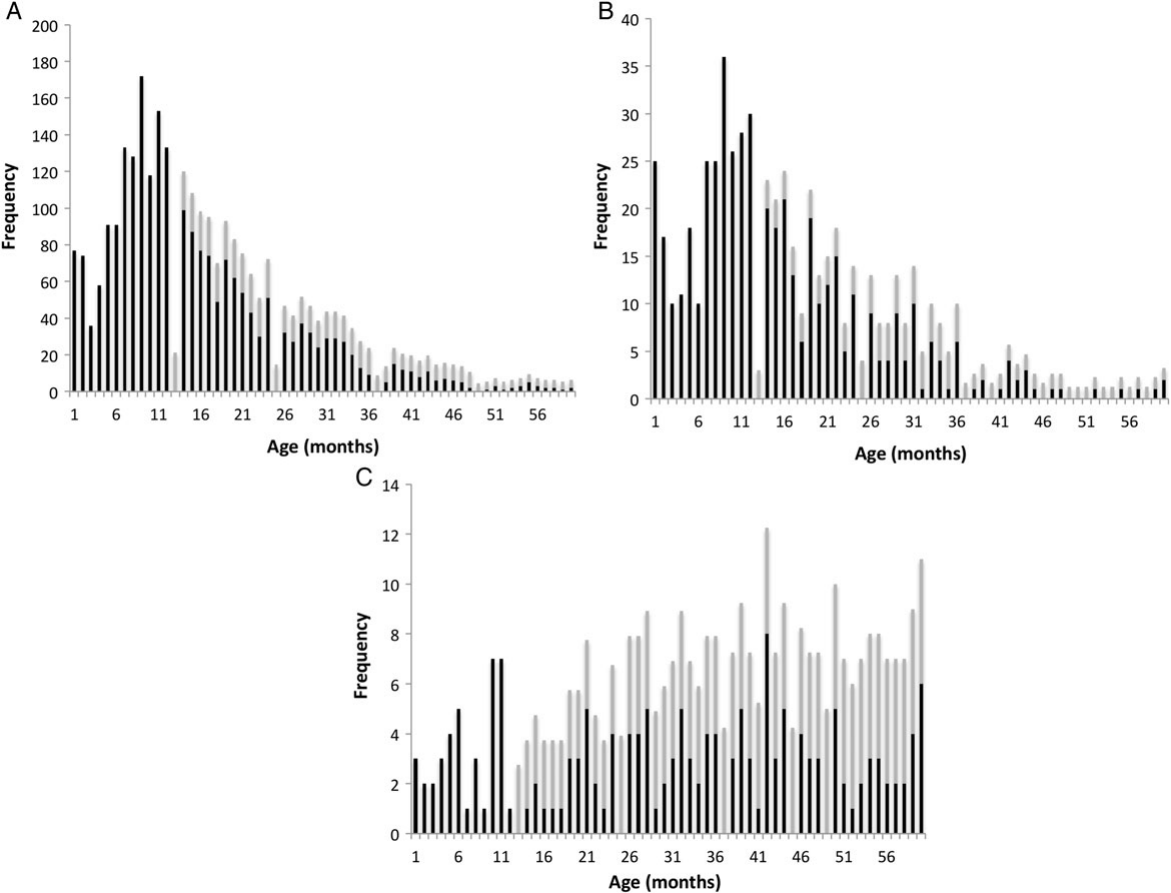
Frequency plots of aggregate age distribution data by month for *Salmonella* Typhimurium (*A*), *Salmonella* Enteritidis (*B*), and *Salmonella* Typhi (*C*) for children <5 years of age. Black bars represent cases where age in months is known and gray bars represent the cases where age is known in years evenly distributed across the year.

Aggregate age distribution data are reported in 4 categories: *Salmonella enterica* serovar Typhimurium, *Salmonella enterica* serovar Enteritidis, *Salmonella enterica* serovar Typhi, and an aggregate of locally untypable serotypes of *Salmonella enterica* species. Data for all 4 groups were reported in 5-year blocks for all age groups (Figure 2) and on a monthly basis for children ≤5 years of age for the 3 most common serotypes (Figure 3). Children <1 year of age routinely had age described in months, but where the age of older children was reported in whole years, the total frequency of cases for that age was equally apportioned across that year of life to smooth the graph—that is, if there were 12 children aged 2 years, 1 case was added to the total for each of months 24–35.

The large numbers of *Salmonella* Typhimurium and *Salmonella* Typhi permit further analysis of trends in age at presentation. Due to the bimodal age distribution of *Salmonella* Typhimurium [11, 12], data were analyzed separately for children <16 years of age and adults ≥16 years. As the age distributions for both the children and adults were nonnormal, the Kruskal–Wallis 1-way analysis of variance was used to screen for variation in age; if significant, an appropriate multiple-comparison post hoc test was used to clarify whether there was a significant long-term increasing or decreasing age trend.

### Inpatient Case Fatality Data

Patient outcome data were only available for selected periods: 2011–2013 for *Salmonella* Typhi, 1999, 2000, 2002–2004, and 2009–2010 for iNTS disease in adults, and 1998 and 2006 for iNTS disease in children (Table 1).

**Table 1.** Temporal Trends in Isolation of *Salmonella* and Estimated Minimum Incidence of *Salmonella* Bloodstream Infection at Queen Elizabeth Central Hospital, Blantyre, Malawi, 1998–2014

Characteristic	1998	1999	2000	2001	2002	2003	2004	2005	2006	2007	2008	2009	2010	2011	2012	2013	2014
Typhoidal *Salmonella* isolated, No.^a^	24	13	5	4	4	9	17	19	18	10	16	19	18	67	186	843	782
Incidence, 100/100 000/y^b^	9.1	4.9	1.9	1.4	1.4	3.1	5.7	6.2	5.7	3.1	4.8	5.5	5	23.4	47.5	206.6	184.1
Case fatality rate, %^c^														3 [20]	3 [20]	3 [20]	
Total nontyphoidal *Salmonella*, No.	541	623	686	783	1381	1323	876	847	671	629	498	343	264	216	154	148	138
*Salmonella* Typhimurium, No.	441	375	397	446	1040	1187	710	737	601	563	443	287	209	185	131	129	119
*Salmonella* Enteritidis, No.	79	209	232	318	226	106	79	78	53	49	42	36	34	22	19	12	13
Other *Salmonella* serovars, No.	21	39	57	19	115	30	87	32	17	17	13	20	21	9	4	7	6
Incidence, 100/100 000/year^b^	103	117	130	137	242	228	147	138	106	97	75	50	37	30	20	19	16
Case fatality rate, %^c^																	
Adults	29 [18]		47 [2]		27 [18]	19 [18]	20 [18]					11 [19]	11 [19]				
Children	23 [3]								20^d^								
Malaria prevalence in febrile children, %				35	33	24	26	17	20	25	26	25	22				
Total No. of blood cultures taken	8545	7823	8052	7653	8978	11 174	10 653	12 933	10 123	9167	8628	7991	8507	9890	10 433	12 815	13 663
Estimated population, Blantyre urban, thousands	527	530	526	571	571	580	596	613	632	645	663	692	721	752	783	816	850

^a^ All *Salmonella* Typhi, no *Salmonella* Paratyphi detected.

^b^ Assumes sensitivity of blood culture 50%.

^c^ Case fatality rate where available from substudies.

^d^ Previously unpublished data.

### Laboratory Methods

Automated blood culture was undertaken at MLW using a standard aerobic bottle (BacT/Alert, bioMérieux, Marcy-L'Etoile, France) since 2000. Prior to this, manual culture was undertaken [13]. CSF samples were cultured at 37°C on sheep blood and chocolate agar for 48 hours under aerobic and microaerophilic conditions. All isolates were identified using standard diagnostic techniques [14]. Salmonellae were identified either by biochemical profile using API 10E or 20E (bioMérieux) or by a combination of their appearance on triple sugar iron agar slant (acid from glucose, no gas, trace or full production of hydrogen sulfide) and a negative urease test. They were serotyped according to the White–Kauffmann–Le Minor scheme by the following antisera: polyvalent O and H, O4, O9, Hd, Hg, Hi, Hm, and Vi antisera (Pro-Lab Diagnostics) [15]. MLW audits blood culture volumes and contamination rates [16] and subscribes to the UK National External Quality Assessment Service (www.ukneqas.org.uk) scheme.

Antimicrobial susceptibility testing was performed by disc diffusion using ampicillin, chloramphenicol, cotrimoxazole, cefpodoxime, and ciprofloxacin discs according to British Society of Antimicrobial Chemotherapy methods and breakpoints. Since 2009, isolates found to be resistant to ciprofloxacin or ceftriaxone by disc testing have had Etests (bioMérieux) performed [17]. Diminished ciprofloxacin susceptibility was classified on the basis of a minimum inhibitory concentration (MIC) between 0.06 mg/L and 1 mg/L) [17]. Isolates were described as “fully susceptible” if susceptible to these 5 antimicrobials and multidrug resistant (MDR) if resistant to ampicillin, cotrimoxazole, and chloramphenicol. Prior to October 2010, these data were entered into ledgers, then double-entered into a validated database. Since then, they have been directly entered into an electronic laboratory information management system.

### Ethics Statement

These data were collected as part of routine surveillance and comply with institutional guidelines.

## RESULTS

Between January 1998 and December 2014, 167 028 blood cultures were taken from adult and pediatric medical patients presenting to QECH (Figure 1). NTS serovars were isolated 10 139 times, of which 8017 (79.1%) were *Salmonella* Typhimurium, 1608 (15.8%) were *Salmonella* Enteritidis, and 514 (5.1%) were not *Salmonella* Typhimurium or *Salmonella* Enteritidis and could not be typed further with locally available antisera. *Salmonella* Typhi was isolated on 2054 occasions, and *Salmonella* Paratyphi A has not been identified in Blantyre.

During the study period, there were 3 epidemics of *Salmonella* BSIs, each caused by a different serovar: *Salmonella* Enteritidis peaked in 2001, *Salmonella* Typhimurium peaked in 2003, and *Salmonella* Typhi peaked in 2013. The number of NTS isolated from blood peaked at 1381 cases in 2002 and declined to 138 cases in 2014, resulting in an incidence of iNTS of 242 per 100 000 population in 2002 and 16 per 100 000 population in 2014. The number of *Salmonella* Typhi isolated from blood peaked at 843 in 2013 for an incidence of 207 per 100 000 population in 2013.

### Age Distribution of *Salmonella* Serovars Isolated From Blood

Supplementary Table 1 and Figure 2 reveal a bimodal age distribution of iNTS disease, which is strikingly similar for *Salmonella* Typhimurium (Figure 2*A*), *Salmonella* Enteritidis (Figure 2*B*), and *Salmonella* species (Figure 2*C*), with peaks under the age of 5 years and between the ages of 20 and 45 years. In contrast, a frequency plot of age distribution of typhoid fever (Figure 2*D*) reveals that it is most common in children and young adults, with a peak age range of 5–10 years.

Analysis of aggregate age distribution data for children ≤5 years of age (Figure 3) reveals a peak of iNTS disease for both *Salmonella* Typhimurium and *Salmonella* Enteritidis in the first 1–2 months of life, prior to a decline at age 2–4 months and then a subsequent climb to a second peak at age 6–12 months (Figure 3*A* and 3*B* and Supplementary Table 2). There is no clear trend in the first year of life for *Salmonella* Typhi (Figure 3*C*).

The 2 serovars responsible for the largest epidemics were investigated for evidence of a significant change in age over time (Supplementary Table 3, Supplementary Figure 1). As *Salmonella* Typhimurium clearly has a bimodal age distribution, median pediatric (<16 years) and median adult (≥16 years) ages were evaluated separately. Supplementary Figure 1*A* reveals that the median age of pediatric iNTS Typhimurium disease has been increasing from a median age of 1.1 years (interquartile range [IQR], 0.7–2.1) at the peak of the epidemic in 2003 to 2.0 (IQR, 1.0–3.0) in 2014. There was evidence of significant variation over time (*P* < .001), and post hoc testing revealed a clear and significant increasing trend in age over time. In the case of adult iNTS Typhimurium disease (Supplementary Figure 1*B*), there was a less obvious change over time, and although there was significant variation in this large data set (*P* < .001) no clear or significant trend was identified. In the case of *Salmonella* Typhi (Supplementary Figure 1*C*), there were too few cases to make a meaningful comparison prior to the onset of the epidemic in 2011. Since then, there has been an apparent fall in median age of presentation. Although the overall variation in age across the *Salmonella* Typhi dataset was significant (*P* = .12), there was no statistically significant trend either for the periods 1998–2014 or the epidemic years 2011–2014.

### Case Fatality

Data were available from 5 published studies and 1 unpublished study on the case fatality rate associated with iNTS disease in adults [2, 18, 19], iNTS disease in children [3], and typhoid fever in both adults and children [20]. Most data were available for adult iNTS disease, which reveals a drop from a peak case fatality rate of 47% observed in 2000, to 11% in 2010 (Table 1). The available data on pediatric case fatality rates are sparser, but suggest a more modest decline from 23% in 2000 to 20% in 2006. Data from the current epidemic of typhoid fever indicate a case fatality rate of 2.5%. The number of annual deaths attributable to iNTS disease and typhoid fever has here been estimated using these hospital case fatality data (Supplementary Table 4) and suggest that in total, blood culture–confirmed iNTS disease caused approximately 2179 inpatient deaths during the study period and typhoid fever caused 51 deaths.

### *Salmonella* Meningitis

In addition to causing BSI, NTS are a common cause of meningitis at QECH (Supplementary Table 5 and Supplementary Figure 2). Between 1998 and 2014, there were 392 cases of culture-confirmed NTS meningitis, 294 (75.0%) due to *Salmonella* Typhimurium, 73 (18.6%) due to *Salmonella* Enteritidis, and 25 (6.4%) due to other NTS. Meningitis caused by *Salmonella* Enteritidis peaked in 2001 and that caused by *Salmonella* Typhimurium peaked in 2002. In contrast, *Salmonella* Typhi has been an uncommon cause of meningitis, with only 9 cases of culture-confirmed *Salmonella* Typhi meningitis.

Nontyphoidal salmonellae were responsible for 23% of cases of culture-confirmed meningitis in children aged 3 months to 5 years between 2000 and 2004, dropping to <10% by 2012; however, they are a less prominent cause of meningitis in older age groups [21]. The case fatality rate is very high in children at 52.3% (average from 1997–2006); however, data are unavailable for adults [22]. Case fatality data are unavailable for the cases of *Salmonella* Typhi meningitis.

### Antimicrobial Resistance

Antimicrobial susceptibility testing was performed on 12 034 *Salmonella* isolates.

#### Multidrug Resistance

**Figure 4.**
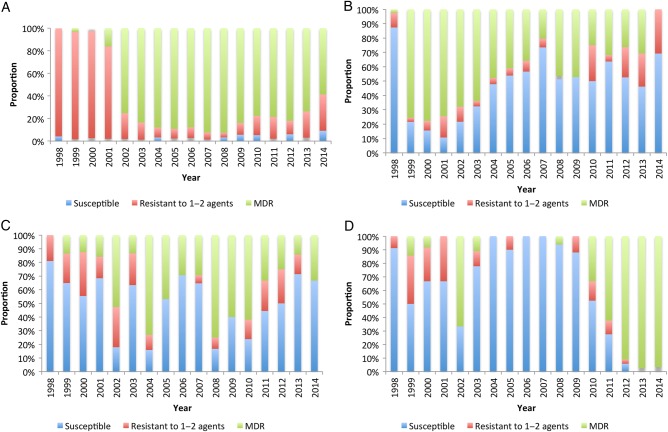
Change in susceptibility pattern to first-line antimicrobial therapy over time for *Salmonella* Typhimurium (*A*), *Salmonella* Enteritidis (*B*), *Salmonella* species (*C*), and *Salmonella* Typhi (*D*). Abbreviation: MDR, multidrug resistant.

The emergence of all 3 epidemics was strongly associated with the MDR phenotype (Figure 4 and Supplementary Table 6). In the case of *Salmonella* Enteritidis (Figure 4*B*), the MDR phenotype subsequently declined from a peak of 78% in 2000, to 0% in 2014, when 0 of 14 isolates were MDR. In the case of *Salmonella* Typhimurium (Figure 4*A*), the MDR phenotype became dominant in 2001 (72% isolates) and was sustained until 2008, when 93% isolates were MDR. Since then, the proportion that was MDR has begun to decline slightly; however, as of 2014, the majority (59%) of isolates remained MDR. Strains of *Salmonella* Typhimurium that were MDR have been found to be part of a novel clade, of multilocus sequence type ST313, with a novel virulence plasmid [23]. In the case of the typhoid fever epidemic (6D), 97% isolates were MDR in 2014. This epidemic is associated with the emergence of an H58-lineage *Salmonella* Typhi in which the multidrug resistance region has inserted itself into the bacterial chromosome on a transposon [20]. In the case of the presumably diverse group of other *Salmonella* species, there was no clear trend (Figure 4*C*) in drug resistance over time. Only a subset of these isolates has been fully serotyped; however, *Salmonella* Paratyphi has not been detected [18].

#### Resistance to Fluoroquinolones and Third-Generation Cephalosporins

In total, 19 isolates have been reported as having altered susceptibility to ciprofloxacin, but only 2 isolates since 2009, when confirmatory MIC testing was introduced: 1 *Salmonella* Typhimurium isolate, which was ciprofloxacin resistant, and 1 *Salmonella* Typhi, which had diminished ciprofloxacin susceptibility confirmed by MIC testing. Sixteen isolates have been reported to be resistant to third-generation cephalosporins, 4 since 2009 (3 *Salmonella* Typhimurium and 1 *Salmonella* Enteritidis). In total, 6 isolates were resistant to both ciprofloxacin and ceftriaxone (Supplementary Table 6), 1 of which, an *Salmonella* Typhimurium isolate from 2009, was further evaluated by whole-genome sequencing and revealed a *CTX-M* gene on an IncHI2 plasmid found in addition to virulence plasmid pSLT-BT [24].

## DISCUSSION

There have been 3 epidemics of *Salmonella* BSI in Blantyre, Malawi, between 1998 and 2014, all of which were associated with multidrug resistance. These findings are mirrored across sub-Saharan Africa, and pan-continental genomic studies have highlighted the role MDR status has played in facilitating the spread of both MDR *Salmonella* Typhimurium ST313 and MDR H58-lineage *Salmonella* Typhi [25, 26]. Although typhoid fever is currently much more commonly diagnosed than iNTS disease, the high case fatality rate for iNTS disease means that all serovars continue to be responsible for a considerable burden of mortality in Blantyre.

We have confirmed the bimodal age distribution of iNTS disease, and additionally demonstrated a relative peak in the first 2 months of life, likely reflecting neonatal or early nosocomial invasive disease. Such data are important for vaccination and prevention strategies. Furthermore, we have demonstrated a gradually increasing median age among children presenting with *Salmonella* Typhimurium BSI.

MDR typhoid fever and iNTS disease are highly prominent causes of hospital admission in Malawi, and are driving the empiric use of third-generation cephalosporins and ciprofloxacin. Although extended spectrum β-lactamase (ESBL)–producing or fluoroquinolone-resistant isolates remain uncommon, they have been detected. Furthermore, the impact of sustained broad-spectrum antimicrobial use on the antimicrobial susceptibility patterns of other Enterobacteriaceae, *Streptococcus pneumoniae*, and *Staphylococcus aureus* is of considerable concern.

### Limitations

These data represent passive hospital-based surveillance from a single center in Blantyre; however, as QECH is the only center in Blantyre where effective treatments are freely available, most patients with MDR invasive *Salmonella* disease are ultimately likely to present to QECH. Some cases will be missed due to deaths in the community, whereas others with drug-susceptible disease may have their disease treated in the community; nonetheless, these are robust estimates of the minimum incidence of invasive *Salmonella* disease in Blantyre.

## CONCLUSIONS

This large longitudinal dataset reveals a dynamic picture of invasive *Salmonella* disease in Blantyre, which emphasizes the importance of country-level surveillance for severe BSI and the pitfalls of extrapolating data across African settings or between different time periods. Invasive *Salmonella* disease in Blantyre displays a complex, changing age distribution and epidemic waves, rather than a static endemic picture, which cannot be attributed to a single risk factor. Sequential epidemics have been associated with MDR, and the first signs of the emergence of ESBL and fluoroquinolone resistance have been seen. The MDR phenotype appears to be a major driver of transmission of invasive *Salmonella* disease, but there is an urgent need to better understand additional factors determining the emergence of different serotypes. There is a particular need for detailed microbiological investigations into potential reservoirs of novel epidemic variants of *Salmonella*, such as *Salmonella* Typhimurium ST313, to understand the transmission of these neglected tropical diseases so that effective vaccine and nonvaccine interventions can be designed.

## Supplementary Data

Supplementary materials are available at *Clinical Infectious Diseases* online (http://cid.oxfordjournals.org). Supplementary materials consist of data provided by the author that are published to benefit the reader. The posted materials are not copyedited. The contents of all supplementary data are the sole responsibility of the authors. Questions or messages regarding errors should be addressed to the author.

Supplementary Data
