# Supplementary material for: Three Epidemics of Invasive Multidrug-Resistant Salmonella Bloodstream Infection in Blantyre, Malawi, 1998–2014
Source: Clin Infect Dis. 2015 Oct 7;61(Suppl 4):S363–71. doi: 10.1093/cid/civ691 (PMC4596930; doi:10.1093/cid/civ691)
Supplement: Supplementary Data [file supp_61_suppl-4_S363__index.html]

Supplementary Data 

# Three Epidemics of Invasive Multidrug-Resistant *Salmonella* Bloodstream Infection in Blantyre, Malawi, 1998–2014

## Supplementary Data

Supplementary Data

- Supplementary Data - Docx file
- Supplementary Tables - xlsx file
