# Supplementary material for: Three Epidemics of Invasive Multidrug-Resistant Salmonella Bloodstream Infection in Blantyre, Malawi, 1998–2014
Source: Clin Infect Dis. 2015 Oct 7;61(Suppl 4):S363–71. doi: 10.1093/cid/civ691 (PMC4596930; doi:10.1093/cid/civ691)

Supplementary Tables

Supplementary Table 1: Aggregate age distribution data for each serotype

Supplementary Table 2: Complete age known and estimated distribution data by month for cases of Salmonella BSI in children <5-years of age at QECH, 1998-2014

Supplementary Table 3: Trends in median age of paediatric iNTS Typhimurium disease, adult iNTS Typhimurium disease and Typhoid Fever

Supplementary Table 4: Estimated deaths per year for blood culture confirmed adult and pediatric iNTS disease and typhoid fever at QECH, 1998-2014

Supplementary Table 5: Complete data on Salmonella meningitis at QECH, 2000-2014

Supplementary Table 6: Complete antimicrobial susceptibility data for Salmonellae isolated from blood at QECH, 1998-2014


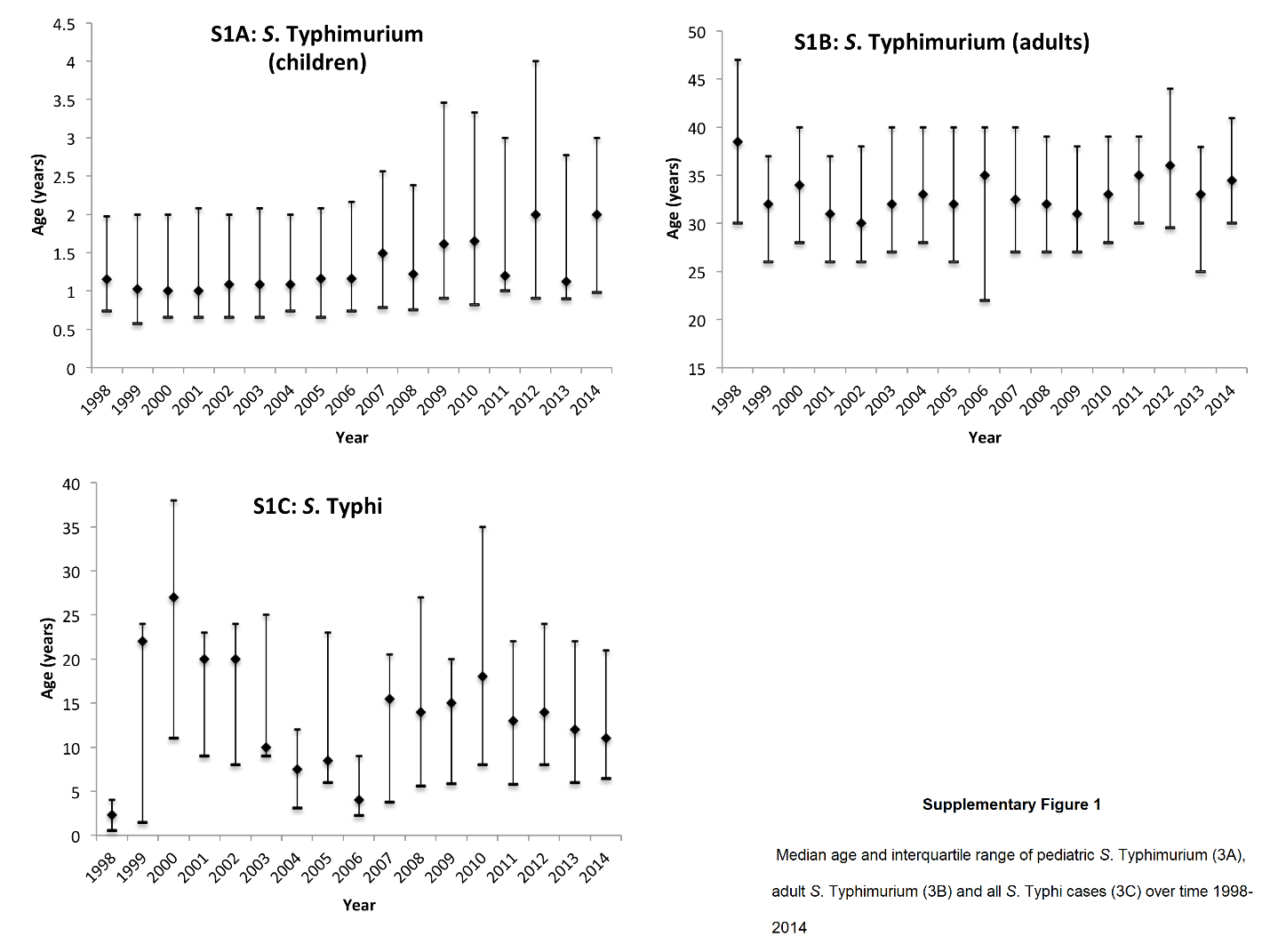


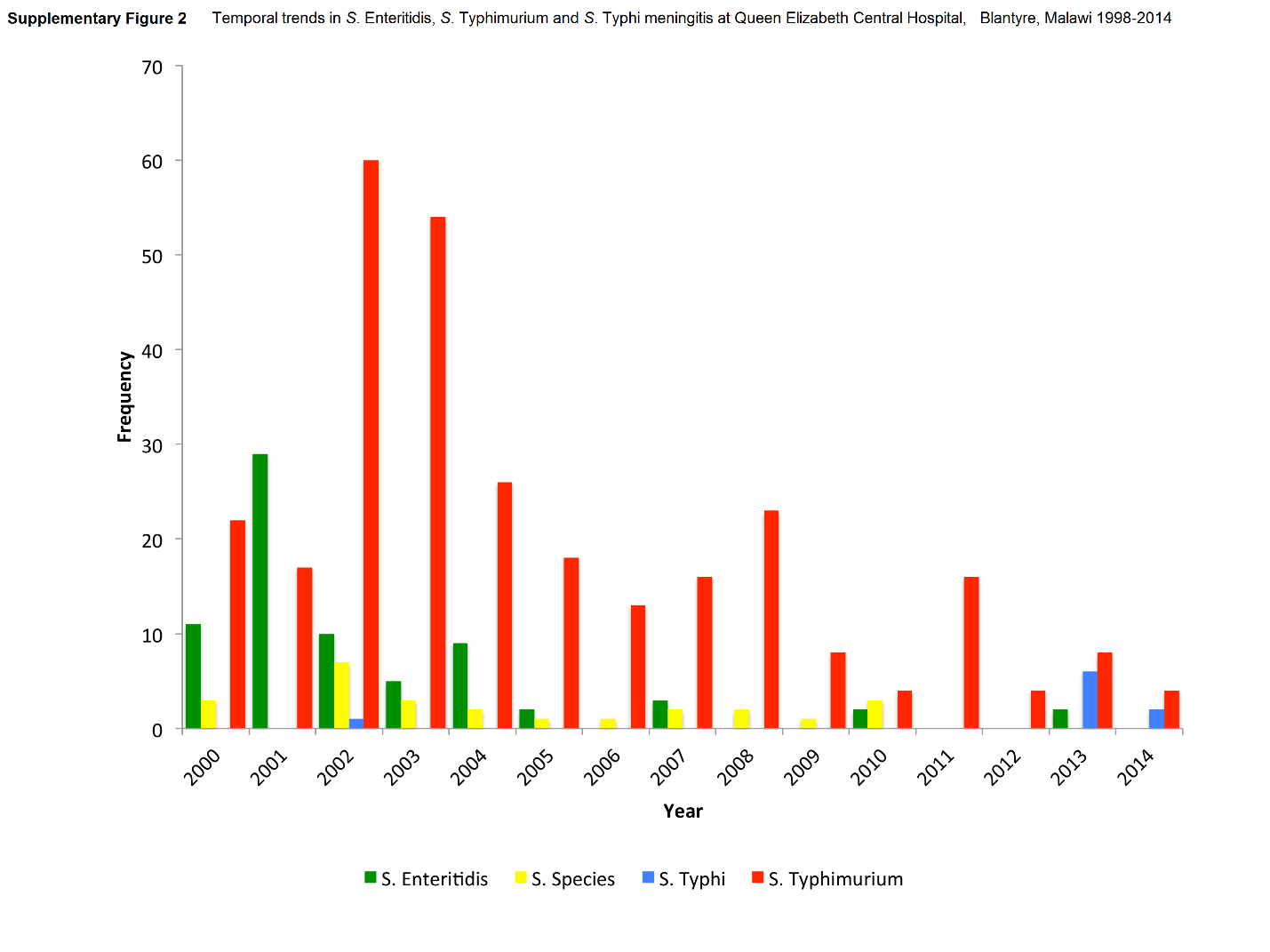

Supplement: Supplementary Data [file supp_civ691_civ691supp.docx]
